# Field Dressings

**Published:** 1900-09-15

**Authors:** 


### FIELD DRESSINGS.

As the result of his experience in South Africa Mr. Lenthal Cheatle[1] says that the cases in which the wounds produced in battle were found to be free from infection were in the minority, "perhaps a large minority." The average of cases free from infection was lowered by the cases from Jacobsdal, Paardeberg, and Osfontein; but even without these the above statement holds good, so that, useful as the first field dressing has proved itself, it will be well to try to render it still more effectual. It is a well-known fact that all wounds in military surgery, or for that matter in civil practice also, which become septic, become infected at the time of their occurrence, or within a few hours afterwards. This is the most critical time of a wound from the point of view of asepsis; hence the importance of improving, if possible, the field dressing which is stitched into the jacket of every soldier's uniform in which he fights. Mr. Lenthal Cheatle points out that this dressing, from the necessities of the case, is almost invariably applied in the absence of any purifying lotion, and that, apart from what the bullet or portion of shell takes into the wound, the patient's skin, fingers and clothes, or the fingers of his dresser, are the chief sources from which infection is derived. Bearing this in mind, he thinks that it would be a great advantage to add to the field dressing a small tube of antiseptic paste, which might be squeezed out upon the finger of the dresser, and on and around the wound, before applying the dressing and bandage. A lotion is out of the question for such a purpose, as also is a puff powder, partly from the difficulty of applying it to the posterior aspect of the trunk and limbs without greatly disturbing the patient, and partly from the fact that in a wind it is blown away. After various experiments Mr. Cheatle recommends a paste of mercury and zinc cyanide, 400 grains; tragacanth in powder, 1 grain; carbolic acid, 40 grains; sterilised water, 800 grains. This should be mixed and made to fill 12 collapsable tubes such as are used for artists' colours. Such a paste can be applied anywhere and in any wind, and in spite of being used in large quantities no signs of poisoning or of local irritation have been observed. The portable field dressing should, then, consist of a collapsable tube containing this paste, double cyanide gauze and double cyanide wool, each in sufficient quantity for two wounds, and a bandage with four safety pins, the whole being enclosed in a mackintosh bag.

[1] Brit. Med. Jour., Sept. 8.

